# An Internet of Things Based Bed-Egress Alerting Paradigm Using Wearable Sensors in Elderly Care Environment

**DOI:** 10.3390/s19112498

**Published:** 2019-05-31

**Authors:** Muhammad Awais, Mohsin Raza, Kamran Ali, Zulfiqar Ali, Muhammad Irfan, Omer Chughtai, Imran Khan, Sunghwan Kim, Masood Ur Rehman

**Affiliations:** 1 Faculty of Medicine and Health, School of Psychology, University of Leeds, Leeds LS2 9JT, UK; m.awais@leeds.ac.uk; 2 Design Engineering and Mathematics Department, Middlesex University, London NW4 4BT, UK; m.raza@mdx.ac.uk (M.R.); k.ali@mdx.ac.uk (K.A.); 3 School of Computing, Ulster University, Newtownabbey BT37 0QB, UK; z.ali@ulster.ac.uk; 4 Electrical Engineering Department, Najran University, Najran 61441, Saudi Arabia; miditta@nu.edu.sa; 5 Department of Electrical and Computer Engineering, COMSATS University Wah Campus, Punjab 47050, Pakistan; omer.chughtai@outlook.com; 6 Department of Electrical Engineering, University of Engineering & Technology, Peshawar 25000, Pakistan; imran_khan@uetpeshawar.edu.pk; 7 School of Electrical Engineering, University of Ulsan, Ulsan 680-749, Korea; 8 School of Engineering, University of Glasgow, Glasgow G12 8QQ, UK

**Keywords:** elderly population, falls, accelerometer, radio-frequency identification (RFID), patient monitoring, Internet of things (IoT), ambulating activities

## Abstract

The lack of healthcare staff and increasing proportions of elderly population is alarming. The traditional means to look after elderly has resulted in 255,000 reported falls (only within UK). This not only resulted in extensive aftercare needs and surgeries (summing up to £4.4 billion) but also in added suffering and increased mortality. In such circumstances, the technology can greatly assist by offering automated solutions for the problem at hand. The proposed work offers an Internet of things (IoT) based patient bed-exit monitoring system in clinical settings, capable of generating a timely response to alert the healthcare workers and elderly by analyzing the wireless data streams, acquired through wearable sensors. This work analyzes two different datasets obtained from divergent families of sensing technologies, i.e., smartphone-based accelerometer and radio frequency identification (RFID) based accelerometer. The findings of the proposed system show good efficacy in monitoring the bed-exit and discriminate other ambulating activities. Furthermore, the proposed work manages to keep the average end-to-end system delay (i.e., communications of sensed data to Data Sink (DS)/Control Center (CC) + machine-based feature extraction and class identification + feedback communications to a relevant healthcare worker/elderly) below $\frac{1}{10}\mathrm{th}$ of a second.

## 1. Introduction

The elderly population is rapidly increasing compared to other age groups [1]. According to the World Health Organization (WHO), the elderly population has increased significantly in the past few decades and is expected to reach 2.1 billion by 2050 [2]. In Europe, the proportion of people aged 65 years or above is foreseen to reach 30% by 2060 [3].

To look after elderly patients, an individual can be chaotic as the patients may move without being noticed (e.g., to leave bed at night without support) and can be potentially vulnerable to hurt themselves (e.g., falling). Accidents such as falls represent a major cause of injury among the elderly. Recent research shows that falls in hospitals are common and cost a lot. In UK, over 200,000 incidents have been reported over 12 months, where falls were the most common safety issue for elderly people [4]. Falls in the hospital caused 26 patients death, 530 hip fractures and about 1000 other fractures reported [5]. The Public Health Outcomes Framework reported 255,000 admissions of patients aged 65 or above in emergency unit of hospitals. Fragility fractures resulted due to the reported falls have been estimated to cost over £4.4 billion, where hip fractures account for nearly £2 billion. In addition, the hip fracture led to an increased one-year mortality of 18% to 33%, while leaving several other negative effects on Activities of Daily Living (ADLs) of suffering individuals [6]. Hospitals, old-age houses, nursing homes, and assistive living conditions also suffer somehow the same circumstances and require additional care towards the movement of the elderly, physically challenged and disabled personnel. Lack of attention, shortage of qualified staff and high dependent to caretaker ratio may result in increased falls. Some of the existing studies based on the recorded data suggest that 51% of total falls occurred while getting into or out of the bed [7]. Unfortunately, the patients/elderly are most vulnerable when waking up from sleep (attempting to leave the bed), as the staff is less attentive towards the sleeping patients. Under such circumstances, it is hard for the staff to ensure safety measures when patients attempt to exit the bed unnoticed. Therefore, it has been an emerging area of research in recent years to counteract and reduce these injuries and fatalities in elderly care environments and clinical settings. Therefore, ongoing research concentrates on the development of accurate human activity recognition methods.

Recent advancements in the miniature motion sensing devices make it possible to monitor the ambulatory activities of elderly population. These sensors are placed on location of interest (wrist, chest, lower back, ankle thigh etc.) [2] to capture the biomechanical movements of the participant by measuring the linear acceleration, angular velocity or both. A typical Inertial Measurement Unit (IMU) consists of a 3D accelerometer, capable of measuring 3D (three axis) linear acceleration and a 3D gyroscope, capable to measure 3D angular velocity. The signals acquired from these IMU sensors are processed further by using the data mining approaches which typically consist of but not limited to; pre-processing, feature extraction, and machine learning. Accelerometers are more commonly used in activity recognition compared to gyroscopes; as these require less battery power to operate than gyroscope, which is a crucial aspect in real life condition. There are various systems developed using accelerometers to classify the physical activities of elderly population. However, these systems mainly focused on laboratory environments with controlled settings. In terms of nature, type, and way of performing activities, a very less is known about the free living ADLs or residential settings [8]. Moreover, the existing systems for elderly population are not validated in real life conditions [9] and such systems developed in controlled environments cannot be translated into real life conditions as their performance degraded significantly when they are exploited in home environments [10]. Therefore, systems developed for elderly people must be validated in the free-living conditions to provide the usability of wearable sensors-based system to monitor the ambulatory ADLs of elderly. More recently, Awais et al. [11] proposed a wearable inertial sensors based activity classification system in which the findings are suggestive of using wearable inertial sensors to accurately classify the ADLs in home environments. Moreover, studies [12,13] showed that wearable sensors can be used to accurately monitor and detect the real world falls with high performance.

Most of the smartphones these days contain built-in 3D accelerometers, which can be effectively used to monitor the ambulation of elderly population. Another system that uses passive Radio frequency identification (RFID), also comes with built-in 3D accelerometers which offers battery-free operation, unlike other systems which require recharging. However, in RFID-based systems, data streams are sparse and often lead to data loss [14]. Therefore, the present work utilizes both datasets (Smartphone and RFID) in different scenarios to develop an IoT-based patient bed-exit monitoring system capable of handling data-streams originating from different sensing modalities.

The use of IoT based healthcare system to monitor patients’ activities is not limited to accelerometers and gyroscope, and several other sensing modalities have been in the past to monitor the patient’s vital signs and provide assistance to lost patients suffering from Alzheimer and dementia. For instance, the authors in proposed a concept of IoT based vital sign monitoring system that can potentially measure heart rate, blood pressure, respiration rate and skin conductance using various sensing modalities; the electrocardiogram (ECG), temperature sensors, galvanic skin response (GSR). Moreover, variety of IoT based fall detection systems are proposed i.e., acoustic fall detection using sound of fall [15], monitoring falls using accelerometers and environmental sensors [16] and combining accelerometers will other sensing modalities such as video cameras and microphone to improve system’s performance [17].

Bed-exit monitoring often mandates real-time observation of the patients in elderly care environment and clinical settings, where the patients are in danger of falling due to pathological conditions and mobility disorders. One important method to reduce falls in care centres is to increase surveillance scenarios, mainly to reduce elderly with a high risk of falls getting up from bed without supervision. If notified in a timely manner, staff can provide required support to the patients and offer supervision. There are various systems developed in this regard to counteract this serious issue [18,19,20,21]. However, there are several limitations in such systems. Some systems use bed mounted sensors [22] to monitor the bed-exit, which limits the applicability of the system to a larger scale where monitoring of other activities such as walking, sitting, and standing might be essential to provide an overall activity profile or sedentary and active behaviour of patients. Moreover, the system developed in [20,21] using RFID does not provide a complete paradigm of patient bed-exit monitoring system in elderly care environment. In most of the earlier works [18,19,20,21], the sensor reading is recorded over time and analysed later, which can be used for identification of ADLs but do not offer a suitable solution for real-time alerting.

The proposed IoT-based communications infrastructure and machine learning based ADLs classification provides a solution that targets real-time data transfer from the sensing devices to DS, its processing and class identification along with feedback communications. All the crucial aspects of paradigm such as communications delays between the device and the DS, dealing multiple patients at a time, computational complexity of bed-exit recognition system, transmission schedule of the devices and alerting mechanisms are modelled to provide a practical solution. The main contributions of the paper are as follows:

(1) A wearable sensors-based patient monitoring system using data mining and machine learning approaches to accurately detect bed-exit.
(2) An IoT-based communications framework to communicate the sensor data to and from the DS/CC.
(3) Assurance of less than 100 ms end-to-end system delay for communications as well as machine classification.
(4) A novel IoT-based bed-exit monitoring paradigm that can timely detect the bed-exit ambulation remotely, and alerts healthcare to take precautionary measures.

The rest of the article is structured as follows: Section 2 presents the system model and dataset used; Section 3, presents results with a comprehensive discussion on findings; Section 4, concludes the study.

## 2. System Model, Materials and Methods

In this work, an IoT enabled infrastructure is proposed to minimize the falls within controlled environments (hospitals, care homes, etc.) as well as uncontrolled environments (private/independent living, etc.). The proposed system embeds a low latency IoT framework for effective communications of sensory information to and from the sensory devices to the Data Sink (DS), where the machine-based analysis is performed to identify ADLs. The proposed work aims to alert the elderly as well as the caretaker of possible transition from one state to the next (e.g., lying to sitting) in near real-time to improve alertness and to avoid falls.

**Figure 1:**
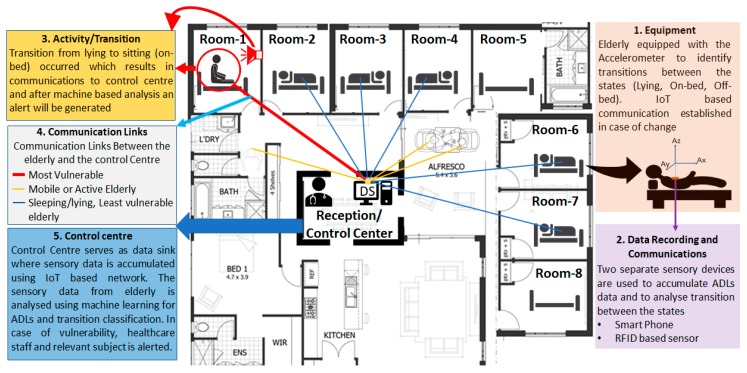
IoT-based patients’ bed-exit monitoring paradigm.

As a proof of concept, two datasets (smartphone—described in Section 2.2.1, RFID—described in Section 2.2.2) are used where the complete infrastructure is proposed to communicate sensory data to the control center, to perform machine-based analysis to classify ADLs and to generate/communicate suitable alerts in near real-time. The block diagram of the proposed system is presented in Figure 1.

### 2.1. Proposed IoT-Based Patient Bed-Exit Monitoring System

The proposed work takes the initial infrastructure of IEEE802.15.4e which schedules communications after regular intervals. However, it is more appropriate to request channel if there is a change in the sensor reading, thus, an ad-hoc solution is proposed which allows devices to request channel based on variations in the sensor reading before each superframe. Channel requests are made prior to communications; therefore, the communication schedule is broadcasted before actual communications take place. On the contrary to Carrier Sense Multiple Access (CSMA) schemes, this limits interference of different devices trying to access the channel at the same time. In addition, for the evaluated case, if the IEEE802.15.4e is used instead of proposed scheme the average delay will increase notably even for low number of requests as the information is to be communicated after regular interval and would also result in unnecessary power consumption.

**Figure 2:**
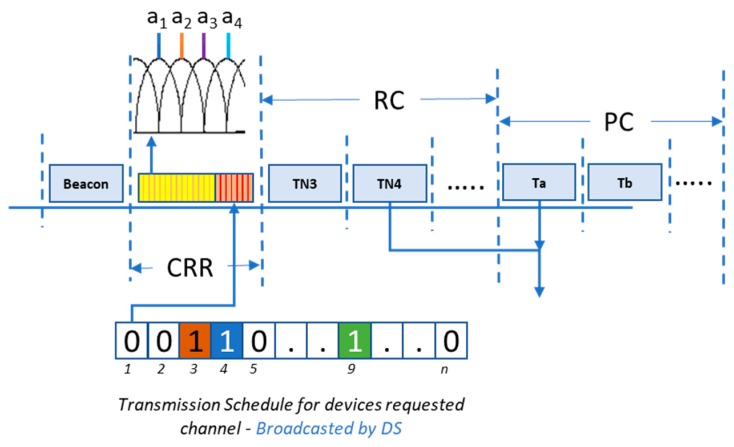
Super-frame structure.

The proposed IoT-based communications infrastructure enables information transfer between the sensing devices and DS. The data processing is performed at the DS to evaluate the ADLs. To ensure real-time communications between the sensing devices and the DS, short super-frame of duration T is proposed [23,24]. A Time Division Multiple Access (TDMA) based transmission is established within the super-frame, where the maximum number of sensing devices affiliated to DS is n, and communication of each sensing device is completed in one timeslot of duration t [25]. Periodic communication schedule is established from the sensing devices to the DS, repeated in regular interval, $T_{R}$ [26,27]. In Figure 2, super-frame structure of the proposed system is presented.

As represented in Figure 2, the super-frame is divided in four subsections: beacon, channel request registration (CRR), requested communications (RC), and periodic communications (PC). Beacon is used to synchronize local clocks of the sensing devices to the DS master clock. CRR allows the sensing devices to request transmission in the current super-frame, when change in sensor readings is greater than the threshold ($\Delta_{th}$). Each of the sensing devices affiliated with DS is allocated a unique orthogonal carrier, which is broadcasted by the sensing devices during CRR. Based on the received carriers in CRR, the DS broadcasts the schedule for RC as represented in Figure 2. RC subsection allows transmission of scheduled communications out of the requested communications during CRR. In PC subsection, the periodic communications from the sensing devices take place. These periodic communications are repeated after interval $T_{R}$. For readers’ convenience, frequently used system parameters are presented in Table 1.

The communication of sensed data from one device takes one timeslot of duration t. Each timeslot is further divided in communication and acknowledgement window of duration (1−δ)×t and δ×t, respectively. Maximum number of sensing devices affiliated to DS is represented by n, where the affiliation process takes place during setup phase in which each device generates an affiliation request to connect to DS.

Total number of communications taking place in a single super-frame is taken as m, where p communications take place during the PC subsection. The communications taking place during the RC are denoted by m−p. Therefore, $T_{R}$ is given by Equation (1).

(1) $$T_{R}=Ceil\left(\frac{n}{p}\right)\times T$$

The communications are scheduled by DS which are followed by the affiliated nodes. Since the number of devices n, can be relatively large thus, to allow the system to ensure near real-time communications, channel can be requested in CRR. The requests are made depending on the variations in sensor values. Given the m−p communication slots available for requested communications, the overall average delay, from communications request to a machine-based decision to control channel-based feedback can be modeled using Poisson distribution where conditional PMF can be given by Equation (2).

(2) $$P_{I}\left(i\right)=\{\begin{array}{c} \frac{\frac{\alpha^{i}e^{-\alpha }}{i!}}{\sum_{j=0}^{n}\frac{\alpha^{j}e^{-\alpha }}{j!}}\;\;where\;j=0,1,\;2,\;\ldots ,\;n\;\\ 0\;\;\;\;otherwise \end{array}$$

The average delay ($d_{avg}$) is modeled as a function of average requests (α), request based communication timeslots (m−p), machine-based processing delay for ADLS (σ), and feedback delay of control channel ($d_{c}$). The mathematical notation for $d_{avg}$ is as follows:

(3) davg=((∑i=1m−pi×αieα/i!∑j=0ni×αjeα/j!)×(m−p2)×t)+ σ+dc+((∑i=m−p+1ni×αieα/x!∑j=0ni×αjeα/j!)×(m−p2)×t+(⌊i(m−p)⌋×T))

Here, machine-based processing delay for ADLS, σ, is evaluated and averaged over several iterations of ADLs identification. σ is dissected into Feature Extraction (FE) and class identification delays. The machine specifications used for the evaluation are presented in Section 2.4. Since $d_{c}$ provides the average delay offered by feedback communications, therefore, system processing delay along with communications scheduling delay are considered. Further details in this regard can be found in Section 3, where $d_{avg}$ is discussed in further detail.

### 2.2. Datasets Analyzed

It is very important to analyze the same population data from which the system is developed as the behavior of ADLs may vary between younger and older population. System’s performance could be degraded, if used among different populations [14]. Therefore, it is advisable to train/develop the system either on the target population’s dataset (if feasible) or a mixture of both (young, older) to maximize the generalizability of the system to work better on both populations.

In the proposed system, we analyzed two elderly subjects’ databases to understand the diverse behaviors of ADLs and to propose a generic system for patient exit monitoring as described in the following subsections.

#### 2.2.1. Smartphone Dataset

**Figure 3:**
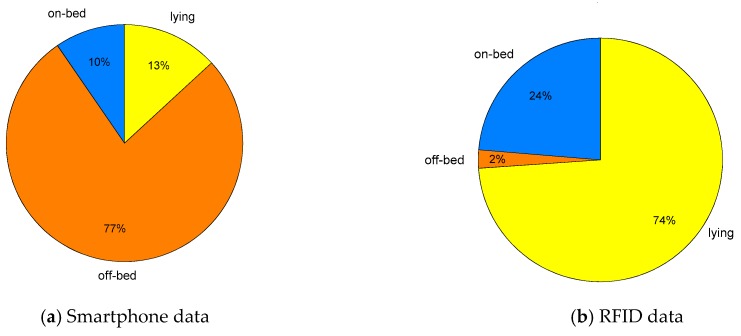
Proportion of data samples in each dataset.

A publicly available dataset is used which is collected using a smartphone-based 3D accelerometer [28] placed inside the front trouser pocket (either on right or left side). Thirty subjects participated in the experiment aged between 18 to 60 years. The dataset recorded smartphone-based 3D accelerometer (A_x_, A_y_, A_z_ are the acceleration signal among *x*, *y* and *z*-axis) readings. The participants performed a variety of ADLs reported in [28], however, only the ADLs related to bed-egress monitoring were analysed. The participants followed a set of protocols describing the sequence of ADLs. Three of the ADLs are relabeled in this work to maintain consistency across datasets. These are as follows: standing up from lying denoted as off-bed, lying down from standing denoted as lying, walking denoted as off-bed. The sampling frequency of 50 Hz is used for data anlysis as this is the commonly used sampling rate for the studies related to human activity recognition [29]. The activity proportion of three aforementioned classes for selected dataset is presented in Figure 3a.

#### 2.2.2. RFID Dataset

Another dataset analyzed for this study is a publicly available dataset [21], collected in an elderly care environment. This dataset comprises of variety of ADLs performed by the elderly population in free living conditions without any structured or sequence of instructions (i.e., performing the ADLs in a natural and unsupervised environment). The RFID sensor measured 3D acceleration (A_x_, A_y_, A_z_) and the received signal strength indicator (RSSI). The RFID sensor is based on Wireless Identification and Sensing Platform (WISP) [30].

The sensor was placed over the sternum to monitor the daily life activities. The activities performed by the subjects were: lying on bed, sitting or transitioning from bed to upright position (on-bed), sitting on chair and ambulating (off-bed). The activity proportion of three aforementioned classes for the selected datasets is presented in Figure 3b. Here, it is worth noticing that the RFID dataset is highly biased towards the lying class as it is the most common ADL performed by the participants, which could be due to any pathological condition, the patient is undergoing.

### 2.3. Feature Extraction from Wearbale Sensors

In machine-based classification, feature extraction plays an important role which directly influences system’s performance. A robust feature-set can help in accurately monitoring and classifying the underlying behavior, while a redundant feature often leads to performance degradation. Therefore, in an initial stage several features were extracted from acceleration signals. While extracting features, it was noted that signal magnitude vector (SMV) (Equation (4)) is very useful while discriminating between sedentary (lying, sitting) and high intensity activities (walking, running) [31]. Therefore, the feature-set comprised of statistical descriptors and time-frequency features were computed from 3D accelerometer and SMV. The complete feature set is listed in Table 2.

(4) $$SMV=\sqrt{A_{x}^{\;\;\;2}+A_{y}^{\;\;\;2}+A_{z}^{\;\;\;2}}$$

Moreover, specific features to RFID were also extracted. It was observed that statistical descriptors computed from RSSI provides distinguishable information about the activity type as the RSSI varies based on distance between receiver and the participant.

**Figure 4:**
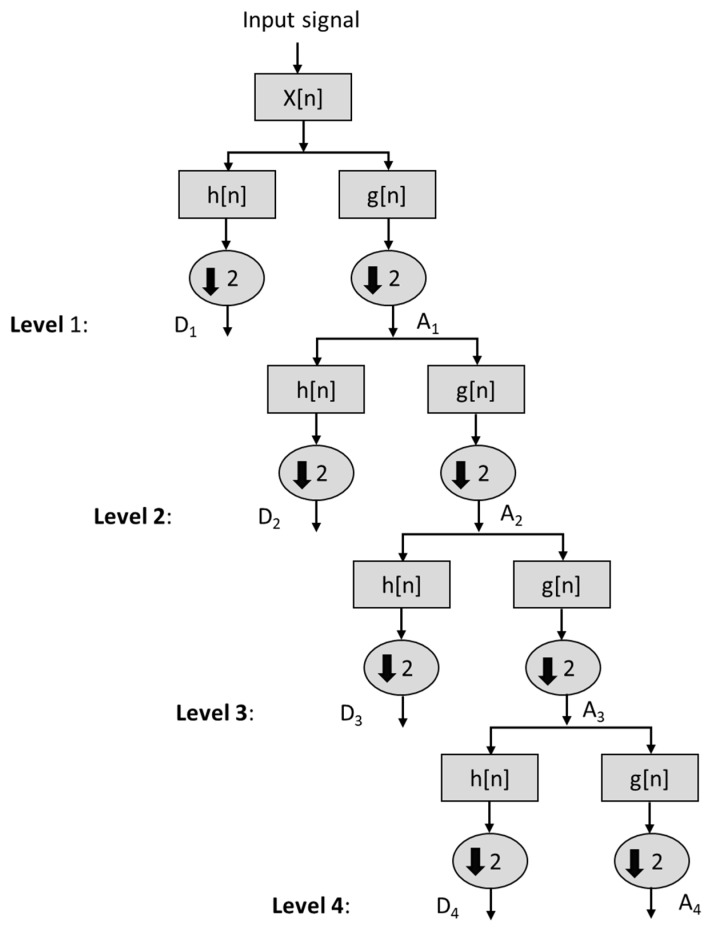
Decomposition of input signal x[n] using DWT with ‘db10’ and up to level 4.

Discrete wavelet transform (DWT) was computed as the time frequency feature from acceleration signal. The Daubechies wavelet ‘db10′ is analyzed in our investigation due to its relevance in discriminating different ADLs [32]. The decomposition levels of input signal x[n] are shown in Figure 4, where the input signal has been decomposed up to 4th level. The signal A*_i_* is the approximate coefficient and D*_i_* is the detailed wavelet coefficient at level i | i =1,2,3 4. The absolute energy (*E_abs_*) and relative energy (*E_rel_*) are computed for each decomposition level (D_1_–D_4_) and at approximation level *A*_4_ [33]. The mathematical expressions for computing *E_abs_*, *E_rel_* and total energy (*E_total_*) are expressed in Equations (5)–(9).

(5) $$E_{abs-D_{i}}=\overset{N}{\underset{j=0}{\sum }}D_{i}\;i=1,\;2,\ldots ,L$$

(6) $$E_{abs-A_{i}}=\overset{N}{\underset{j=0}{\sum }}A_{i}\;i=L$$

(7) $$E_{total}=\overset{L}{\underset{i=1}{\sum }}E_{abs-D_{i}}\;+\;E_{abs-A_{L}}$$

(8) $$E_{rel-D_{i}}=\frac{E_{abs-D_{i}}}{E_{total}}\;i=1,\;2,\ldots ,L$$

(9) $$E_{rel-A_{i}}=\frac{E_{abs-A_{i}}}{E_{total}}\;i=L$$

where *L* is the total number decomposed, and *N* is the length of input signal. A total of ten features are computed from DWT corresponding to each signal.

A time window of 151 (3.02 s) samples is used to compute each feature from Smartphone data. It is worth mentioning that the DWT features are computed only for the smartphone, since the smartphone data is uniformly sampled at 50 Hz, while RIFD data streams are sparse and non-uniform due to the passive nature of communications [30]. Thus, a reliable derivation of the frequency domain feature for raw RFID sensor data is not possible. Statistical description of time domain features and other features listed in Table 2 are computed across a time window of 50 samples. The ground truth information (start and end of each ADL with respect to sensor’s data) is provided within both datasets.

### 2.4. Machine Learning and Performance Evaluation

Two different machine learning algorithms were implemented to observe the proposed system performance using defined datasets. The different classifiers applied in the system are Random Forest (RF) and Support Vector Machine (SVM), as these are the most commonly used classifiers which perform quite well on time series wearable sensors’ data [34,35]. We also implemented a variant of SVM i.e., weighted SVM (W-SVM) by assigning different weights to each class in order to penalize the majority class. Introducing penalty could be effective especially when dealing with unbalanced datasets, which is quite often the case in real life conditions [11]. We utilized the method proposed by Huang et al. [36] to compute the weights of each class by dividing the majority class samples to each of the classes. For example: N_1_ = 200, N_2_ = 300, N_3_ = 2000 are the epochs (time windows) of three classes, then the weights of each class would be: W = [W1, W2, W3 = [N_max/_N_1_,N_max_/N_2_,N_max_/N_3_] where N_max_ is majority class, i.e., N_3_ in this example. The penalized system model was then developed by assigning the derived weights to the W-SVM classifier. The machine learning algorithm are implemented in MATLAB.

The F-Score is computed as a performance measure to analyze the overall performance of the proposed system as well as performance by class of different activities on both datasets. The expression used to calculate F-Score by class is expressed in Equation (10). The overall performance is computed by taking the average performance of all classes.

(10) $$F-Score=\frac{2\times TP}{2\times TP+FP+FN}\times 100$$

where *TP* = True Positive (correctly classified instances of the selected class which is also the actual class), *FN* = False Negative (incorrectly classified instances of a selected class to another class which is not the actual class), *FP* = False Positive (incorrectly classified instances which belong to another class than the selected class). The selected class is the class classified by the proposed system, while the actual class is the class obtained through ground truth data, either by visual inspection of a human observer during data collection or by annotating the video recording of data collection sessions.

The performance of the proposed system is computed using 5-fold cross validation procedure, where 80% of the data is used to train the classifier and remaining 20% is used to compute system performance. The samples distribution in both sensing modalities during 5-fold cross validation is as follows; 80% of Smartphone’ data split (training data) contains 1800 samples (each of 5 s), 80% of RFID’s data split (training data) contains 1091 samples (each of 5 s), 20% of Smartphone’ data split (testing data) contains 450 samples (each of 5 s) and 20% of RFID’ data split (testing data) contains 273 samples (each of 5 s). The number of features are; 91 and 52 (see Table 2) for Smartphone and RFID respectively, which are then fed to SVM and RF classifiers to compute system’s performance. This procedure is repeated five times to compute the performance using all dataset.

We also performed the computational complexity analysis of both systems (Smartphone, RFID) by measuring the time required to compute all features for a single time window and the time required to classify (test) a single window instance by all classification algorithms. The computational complexity is then scaled to four, as if we’ve received four traces (four data streams from four patients) at a time. This process is repeated ten times to account for the PC variability; and then average and standard deviation is computed. The simulations were performed on Intel(R), Core (TM) i7-6700 CPU @3.4GHz, 8.00 GB RAM.

## 3. Results and Discussion

### 3.1. Performance Evaluation of Proposed System on Different Datasets

#### 3.1.1. Smartphone Data Analysis

The overall performance achieved using three different classifiers on Smartphone’s dataset is presented in Table 3. The respective confusion matrices of all three classification scenarios for smartphone are listed in Table 4. All three classifiers achieved an overall performance of 90% which is very encouraging and shows potential of the proposed system in patient bed-exit monitoring. However, the performance by class of on-bed and lying is significantly lower than the off-bed class for RF and SVM classifier as depicted in Figure 4. The rationale behind this is that the off-bed activity is of very high intensity as it contains walking and locomotion activities and their signal energy is much more than the other sedentary ADLs on-bed and lying. An effort has been made in feature extraction stage to counteract by deriving the orientation feature of Smartphone (i.e., the mean of each axis acceleration) [37]. However, this issue persisted due to the class imbalance, which is evident from Figure 3a that the off-bed class contains more than three quarters of the total dataset samples and the remaining two classes (on-bed and lying) contain less than one fourth.

**Figure 5:**
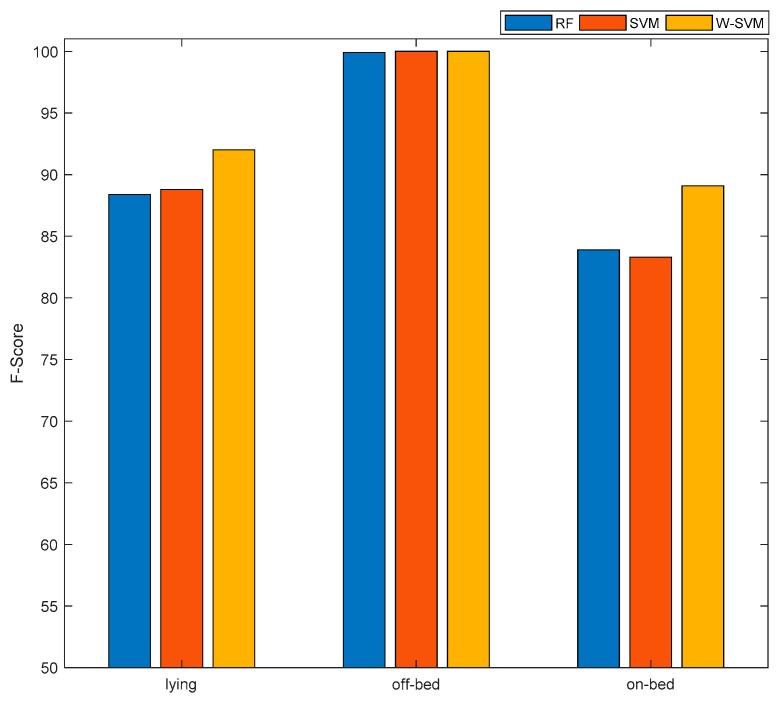
Performance analysis of Smartphone data on different Activities of Daily Livings (ADLs) using various classifiers.

An effort has been made to tackle the classification bias occurred due to class imbalance by using the weighted SVM (W-SVM). The findings show that W-SVM notably improves the overall performance of the system as well as the performance of the underrepresented classes as highlighted in Table 3 and Figure 5 (Smartphone, W-SVM). The improvement in performance, while moving from SVM to W-SVM is 5.8% in on-bed class, 3.6% in lying class and 3% in overall performance of the system. These findings show the significance of using W-SVM in the scenarios where majority represented class is penalized by assigning lower weights as compared to underrepresented classes. Such system model has implications in real life conditions where it is quite often the case that one or set of ADLs are overly represented in the datasets compared to others.

The off-bed class performed the best (100%) among all classes with second highest performance achieved by lying class (92%). On-bed class had relatively lower performance (89.1%) than the rest of classes. Still, the performance of on-bed class is quite acceptable as this is an important aspect of the proposed system to classify on-bed class (lying to standing transition) with high F-Score (89.1%). This is because, it is quite essential to track the off-bed attempt of a patient in order to provide an early notification to caregiver and healthcare staff to take precautionary measures beforehand.

The computational complexity of feature extraction time and testing time of four traces is presented in Table 5. It is evident from the results that the total computation time (sum of feature computation and testing) is quite less and optimal (less than 5msec) to classify four traces in real-time.

#### 3.1.2. RFID Data Analysis

The performance achieved using RFID dataset is reported in Table 6 and the respective confusion matrices are listed in Table 7. The best overall performance is achieved using the RF classifier (88.4%), however the difference in performance between RF and SVM is negligible (0.3%). These findings show that any of these classifiers can be a viable solution to detect bed-exit with an acceptable level of performance. However, contradictory to Smartphone findings (Section 3.1.1), the W-SVM classifier has degraded the system performance instead of improving it, which is in-line with the findings of [11] suggesting that weighted classifiers are not always useful to improve the performance especially in scenarios where the samples of underrepresented class are much lower than other classes, thus highlighting the need for collecting more samples.

**Figure 6:**
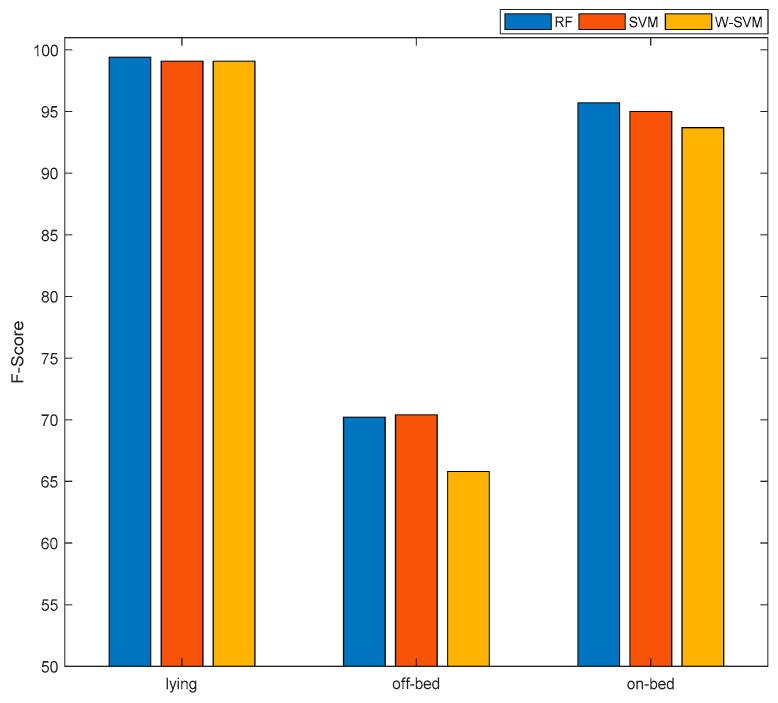
Performance analysis of RFID data on different ADLs using various classifiers.

The performance of off-bed class is lowest among all classes. The maximum performance achieved by off-bed class is 70%. The performance of on-bed and lying classes is above 95% (see Figure 6). The possible reason behind this low performance and low sample size is the quality of data recorded and communication ability of RFID sensors, which can be improved using IoT enabled solutions. It is worth mentioning that the analysis performed in the current work is assuming no data loss during data collection, as if it is stored at a uniform sampling rate. However, this is not the normal practice when dealing with RFID based sensors as the noise and sparse nature of these devices affects data streams, resulting in significant data loss. This behavior is also justified from the findings of the present work suggesting that the highly affected class is the off-bed which is a combination of walking and locomotion ADLs. Therefore, as the patient moves from one location to another, the signal to noise ratio (SNR) is significantly degraded as the distances between the transmitter and receiver varies recurrently, resulting in much higher packet loss in locomotion ADLs (walking, moving) than those of sedentary ADLs (off-bed, lying).

Nevertheless, the scope of present work is to propose a generic paradigm of elderly monitoring in clinical environment or nursing homes which can effectively recognize the lying and on-bed movements where the proposed systems achieves relatively high accuracy by class for these ADLs (Figure 6).

### 3.2. Implications of IoT Based Patient Monitoring System in Healthcare

The primary objective of the proposed IoT based machine intelligence solution is to identify the potentially alerting movements in elderly to notify the healthcare staff as well as caution the elderly. For the proposed system to be effective, false alarms need to be minimized for which high accuracy of machine learning is of utmost importance. Thus, a high accuracy machine intelligent system is proposed. In addition, this system needs to work in near real-time to not only communicate the sensed information to DS, but to also perform machine analysis and issue alerts, if needed. Therefore, along with the proposed high-performance machine classification system, this section discusses the overall system delay from initiation of request to the final feedback.

**Figure 7:**
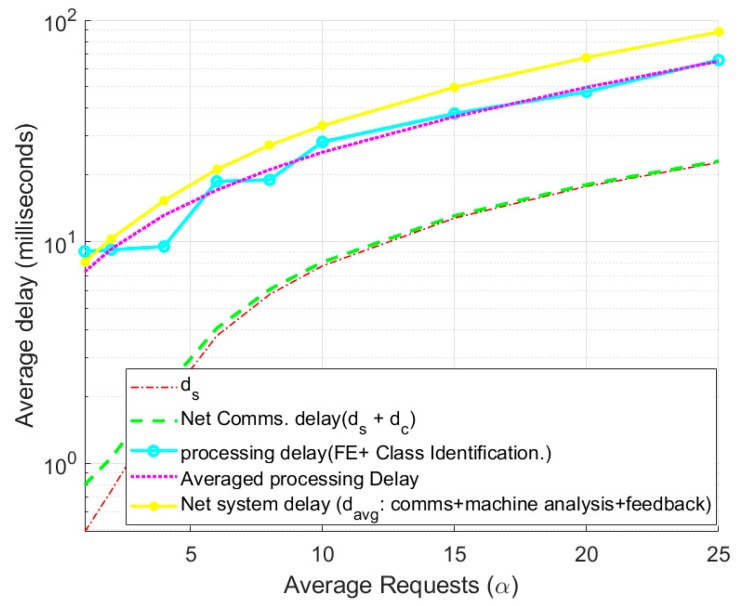
Communications, processing and System delay.

In Figure 7, the overall system delay is presented as a function of average requests initiated from the sensing devices. As explained earlier, the requests generated by the sensing devices depends on the sensor readings and only if notable change is detected in sensing value, request is generated. In idle state, the sensing device will communicate its data to DS after a regular interval, $T_{R}$.

Averaged sensed data communications delay ($d_{s}$), which is the delay when a sensing device request for channel to its communication to the DS, is evaluated to be ranging from 500 μs to 18 ms for varying sensing devices’ requests (α=1 to 25). Even for the relatively high channel requests (α=25), the offered delay is well within the limits. For the performed simulations, the delay in case of IEEE802.15.4e will remain consistent as represented in Figure 7. In addition, any sensory device will not be able to communicate earlier than the specified periodicity of communication selected initially.

As a comparison, between the proposed IoT framework and IEEE802.15.4e, for an average 10% of elderly being active (i.e., 10% of the sensory values are changing) average requests per frame will be 5 for n=50, thus only 5 requests will be made resulting up to 3 ms delay in comparison to 25 ms delay needed in case of IEEE802.15.4e.

In addition, since the machine based analysis allows multi-threading with up to ω parallel streams, so the added delay for control channel based feedback communications is relatively low and can range from t to ω×t. Average processing delay for machine based analysis including Feature Extraction (FE) and class processing ranges from 9 ms to 76 ms for average requests from 1 to 25 respectively. Taking all the delays in consideration (i.e., average end-to-end delay), the proposed system generates alerts within $\frac{1}{10}\mathrm{th}$ of a second, allowing in time analysis and feedback for reduced vulnerability in elderly and physically challenged personnel.

## 4. Conclusions

This paper proposed an applicable solution for governance and artificial alertness in monitoring the elderly population and patients with mobility disorders. The proposed framework uses both IoT-based communications and DS-based machine-learning/classification to identify ADLs, performed by elderly, to highlight potentially alarming activities and alert the relevant healthcare staff. The proposed work offers a relatively high accuracy in classification of ADLs, especially, during the bed-exit, which is a leading cause for most of the reported falls. The system also managed to minimize the end-to-end delay, where any potential alarming state transition is classified and reported to the relevant personnel in $\frac{1}{10}\mathrm{th}$ of a second. The proposed work is very practical and suitable for the healthcare centers, hospitals, old age houses with centralized processing unit (in our case labelled as DS), offering rapid processing and enabling timely alerts. However, for remote use and in households, the work can be further improved with the inclusion of edge and fog computing, with wireless/wired backhaul to a centralized cloud.

The future work should emphasize on validating and testing the proposed system in real life conditions in a clinical environment or residential settings to fully exploit its applicability of alerting healthcare staff by generating alarms in off-bed movements of elderly, and to detect and prevent falls. Moreover, this will fine tune the proposed method to perform well in clinical and residential settings by addressing the possible limitations that can occur in validation stage.

## Figures and Tables

**Table 1:** System Parameters, symbols and values.

Parameters	Variables	Value(s)
Superframe Duration	T	5 ms
Periodic communications interval	$T_{R}$	-
Total Sensing devices affiliated to DS	n	50
Total Timeslots in superframe	m	10
Timeslots in PC	p	-
Timeslots in CRR	m−p	-
Timeslot duration	t	~300 µs
Communications duration in timeslot	(1−δ)×t	~250 µs
Communications duration in timeslot	(δ)×t	~50 µs
Average delay from request to feedback	$d_{avg}$	-
Averaged sensed data communications delay	$d_{s}$	
Control channel-based feedback delay	$d_{c}$	-
Machine-based processing delay	σ	-
Parallel Processes	ω	4
Sensor reading threshold	$\Delta_{th}$	-
Average requests	α	0, 1, 2 … n/2

**Table 2:** Features computed from different signals and datasets.

Feature	Smartphone Dataset				RFID Dataset				
	x	y	z	SMV	x	y	z	SMV	RSSI
Mean	✔	✔	✔	✔	✔	✔	✔	✔	✔
STD	✔	✔	✔	✔	✔	✔	✔	✔	✔
RMS	✔	✔	✔	✔	✔	✔	✔	✔	✔
Minimum	✔	✔	✔	✔	✔	✔	✔	✔	✔
Maximum	✔	✔	✔	✔	✔	✔	✔	✔	✔
Range	✔	✔	✔	✔	✔	✔	✔	✔	✔
Zero crossing	✔	✔	✔	✔	✔	✔	✔	✔	✖
Correlation	✔	✔	✔	✖	✔	✔	✔	✖	✖
P_25th_	✔	✔	✔	✔	✔	✔	✔	✔	✖
P_75th_	✔	✔	✔	✔	✔	✔	✔	✔	✖
Energy P_25th_	✔	✔	✔	✔	✔	✔	✔	✔	✖
Energy P_75th_	✔	✔	✔	✔	✔	✔	✔	✔	✖
Wavelet Energy	✔	✔	✔	✔	✖	✖	✖	✖	✖
Total features	91				52				

*Correlation*—computed among accelerometer axis resulted in 3 features i.e., (x,y), (x,z),(x,z); *STD*—standard deviation; *RMS*—root means square; *P_25th_*—25th percentile, *P_75th_*—75th percentile; *Energy P_25th_*—sum of squared elements below P_25th_; *Energy P_75th_*—sum of squared elements below *P_75th_*; ✔—feature computed from the respective signal; ✖—feature not computed from the respective signal.

**Table 3:** Overall performance and performance by class using different classifiers on Smartphone data.

Classifier	Smartphone Data			
	On-Bed	Off-Bed	Lying	Overall
RF	83.9	99.9	88.4	90.7
SVM	83.3	100.0	88.8	90.7
W-SVM	89.1	100.0	92.0	93.7

**Table 4:** Smartphone results using different classification algorithms (**a**) Random Forest, (**b**) SVM, (**c**) W-SVM.

	**(a) RF**			
	**Predicted Class**			
**Actual Class**	**on-bed**	**off-bed**	**Lying**	**←classified as**
	180	2	34	**on-bed**
	0	1737	1	**off-bed**
	33	1	262	**lying**
	**(b) SVM**			
	**Predicted Class**			
**Actual Class**	**on-bed**	**off-bed**	**lying**	**←classified as**
	170	0	46	**on-bed**
	0	1738	0	**off-bed**
	22	1	273	**lying**
	**(c) W-SVM**			
	**Predicted Class**			
**Actual Class**	**on-bed**	**off-bed**	**lying**	**←classified as**
	193	0	23	**on-bed**
	0	1738	0	**off-bed**
	24	0	272	**lying**

**Table 5:** Computational complexity across different classifiers for four traces received at a time.

Dataset	Feature Extraction Time (ms)	RF Testing Time (ms)	SVM Testing Time (ms)	W-SVM Testing Time (ms)
Smartphone	4.13 ± 1.55	0.41 ± 0.03	0.39 ± 0.04	0.64 ± 0.11
RFID	3.15 ± 0.55	4.77 ± 0.80	0.14 ± 0.02	0.14 ± 0.01

**Table 6:** Overall performance and performance by class using different classifiers on RFID dataset.

Classifier	RFID Data			
	On-Bed	Off-Bed	Lying	Overall
RF	95.7	70.2	99.4	88.4
SVM	95.0	70.4	99.1	88.1
W-SVM	93.7	65.8	99.1	86.2

**Table 7:** RFID results using different classification algorithms (**a**) Random Forest, (**b**) SVM, (**c**) W-SVM.

	**(a) RF**			
	**Predicted Class**			
**Actual Class**	**on-bed**	**off-bed**	**lying**	**←classified as**
	312	3	8	**on-bed**
	13	20	0	**off-bed**
	4	1	1003	**lying**
	**(b) SVM**			
	**Predicted Class**			
**Actual Class**	**on-bed**	**off-bed**	**lying**	**←classified as**
	311	2	10	**on-bed**
	13	19	1	**off-bed**
	8	0	1000	**lying**
	**(c) W-SVM**			
	**Predicted Class**			
**Actual Class**	**on-bed**	**off-bed**	**lying**	**←classified as**
	303	15	5	**on-bed**
	9	24	0	**off-bed**
	12	1	995	**lying**
